# Relationship between Objective and Subjective Atmospheric Visibility and Its Influence on Willingness to Accept or Pay in China

**DOI:** 10.1371/journal.pone.0139495

**Published:** 2015-10-07

**Authors:** Kangkang Yu, Zhenghao Chen, Jian Gao, Yuechong Zhang, Shulan Wang, Fahe Chai

**Affiliations:** 1 School of Agricultural Economics and Rural Development, Renmin University of China, Beijing, P.R. China; 2 State Key Laboratory of Environmental Criteria and Risk Assessment, Chinese Research Academy of Environmental Sciences, Beijing, P.R. China; 3 Collaborative innovation center of atmospheric environment and equipment technology, Nanjing, Jiangsu, P.R. China; Nanjing University, CHINA

## Abstract

This study is to distinguish the objective and subjective measures of atmospheric visibility, and investigate the relationship between the two measures as well as the effect on the people’s behavioral intentions on air pollution in China. A mixed method was adopted in this study combining both lab experiments to measure objective atmospheric visibility and a questionnaire survey to measure subjective atmospheric visibility. The regression results show that: (a) The people’s perception of atmospheric visibility is based on objective information about the ambient air (Relative Humidity, PM_2.5_, Atmospheric Visibility) and there are some turning points that could enable people to distinguish good and poor air quality; (b) The people’s perception of visibility has a significant effect on either their willingness-to-accept (WTA) the visibility or on their willingness-to-pay (WTP) for improving the air quality; (c) The subjective atmospheric visibility also mediates the effect of objective measures on WTA or WTP; (d) The higher the level of pro-environmental attitude is, the more people will pay to improve the air quality, and this effect is much stronger than that effect of perception; and (e) The respondents from North China Plain, Yangtze River Delta and the relative clean areas have higher level of perception.

## Introduction

China has experienced a huge economic growth in the past three decades, with an annual growth rate of 10% for the gross domestic product, and the remarkable growth of its economy has resulted in increased energy consumption as well as air pollution and visibility damnification[1]. Atmospheric Visibility is one indicator for measuring air quality and also one of the most significant factors in evaluating air pollution [2]. The Visual Air Quality (VAQ) is often estimated by Light Extinction (Bext), Deciview, Visibility (Vis) and Particulate Matter (PM_2.5_). Evaluation of the relationship between PMs and Bext, is critical in establishing control measures to alleviate visibility degradation [3–6]. Compared with Bext, the haze index and visibility show a linear relationship [7,8].

Researchers used to use the pictures simulated by computer programs or a series of real photos to describe the visibility of the city view in a certain region in order to evaluate the level of the people’s assessment and acceptance of the visual air quality [9–11]. In terms of the former method, software called WinHaze is often adopted to finally generate digital photos, while the latter picks up real photos according to a few indicators. For example, in the report about people perceptual visibility, Davidson et al [12] asked the respondents to evaluate several photos showing the visibility in Washington D.C., according to their attitude toward the visual air quality in each of the assigned photos. The relationship between VAQ/PM and acceptance were then analyzed from the results. The level of acceptance is usually measured by a Contingent Valuation Method (CVM). In terms of the research on visibility, having shown the respondents a series of photos of the same place with different levels of visibility, the researchers position them in a certain context in which the visibility has been changed to see if they think that it is acceptable and if so, how much they would be willing to pay for producing such a change. For example, in the early 1980s, Schulze et al [13]estimated how much all the national parks in the southern and western parts of America would have to spend to avoid a reduction in visibility, and some researchers have attempted to conduct small-scale studies to address similar problems [14,15]. These studies not only have geographical limitations, but also only evaluate the change in visibility at a single level; therefore, it is doubtful that these results could be used in other places, which would constrain the generalizability. In order to address this problem, Rae [16] expanded the research scope to the benefits brought by reducing air pollution in urban areas, although he fails to stipulate the absolute value of the change in visual air quality. In some of the aforementioned studies, the respondents are not in the real context, while other studies evaluate the effect of visibility on tourists during their visit to national parks [17–19]. However, this kind of research is also biased because tourists may not only spend money to enjoy the beautiful views, but on other tourism amenities.

Although the early researchers in the fields of environment science and environment management have tried to build a bridge between visual air quality and the people’s behavioral intentions, it is not easy to draw a direct linkage between objective atmospheric visibility and human behaviors. Apart from objective measures, some studies in the field of environmental psychology have also begun to focus on the people’s perception of visibility and assessment on the basis of this perception. In terms of visual landscape assessment, human perception is the core of the definition that “an area, as perceived by people, whose character is the result of the action and interaction of natural and /or human factors” [20]. The distinction between the objectivist approach, in which the visual quality is viewed as being inherent to the ambient air, and the subjectivist approach, in which the visual air quality is considered to be constructed by the observer [21], is parallel to the long-standing debate about whether beauty is “in the object” or “in the eye of the beholder” in the philosophy of aesthetics [22].

Similar to landscape assessment, we argue that there are some differences between the visibility level perceived by the people and the level calculated based on lab experiments. The key questions are why there are differences and how they would affect the people’s behavior. So far, there have been no good answers to these questions. While environmental psychology, which focuses on the relationship between humans and the natural environment, has been the basis of much of the research on the people’s cognition of the environment and their behaviors toward it, only a few of these studies combine both an objective and subjective perspective. In this study, we adopt both objective atmospheric visibility based on lab experiments and subjective atmospheric visibility based on a questionnaire survey in order to investigate the relationship between these two measures as well as its effect on the people’s behavioral intentions.

## Methods

A mixed method was adopted in this study. As explained earlier, we collected objective data by means of experiments and subjective data by conducting a survey. This study has been approved by Renmin University of China and the data were analyzed anonymously. The participants provide their online informed consent to participate in this study. If they agree with the instruction in the consent, they will finish the online questionnaire. However, if they do not agree with it, they can drop up participating in the survey. We have downloaded all the questionnaires online together with the instruction on the first page. All these copies have been documented in a hard disk by the research team. Furthermore, the ethic committee approved this procedure. The processes of developing the measure and collecting the data are explained in the following subsection.

### Objective Measures

In studies of visibility, researchers who adopt a contingent valuation method (CVM) would normally show the respondents a series of photos that represent different levels of visibility in the same place. In this study, the photos shown to the respondents were chosen from those taken in October 2011 on the roof of the observation station in the campus of the Chinese Research Academy of Environmental Sciences, which is located near a residential area in the northern part of Beijing (116°24’E, 40°02’N) outside the fifth ring road of the city. The distance from the sampling site to the 2008 Olympic Games stadium is only 5.7 km and the Olympic village is less than 3 km away. The instruments were set up in a three-room observational station located on the rooftop of a three-tier building [23].

**Fig 1:**
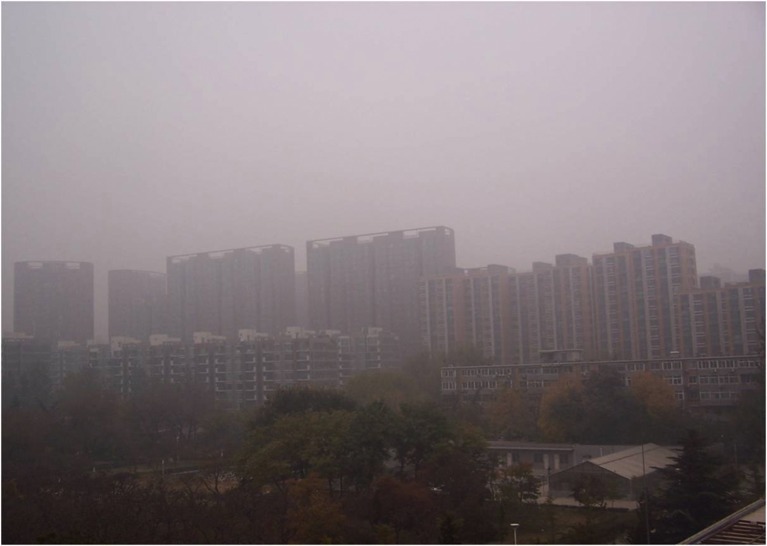
Photo No.1 in CVM (Haze Degree: Severe, RH = 83.5%, PM2.5 = 170.6μg/m3, PM10 = 411.0μg/m3, Vis = 1.6 km, AQI = 461.

**Fig 2:**
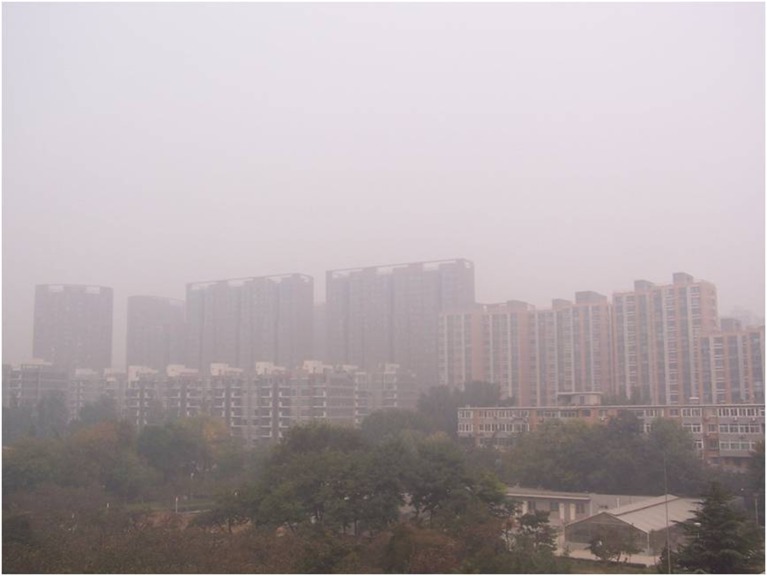
Photo No.2 in CVM (Haze Degree: Moderate, RH = 69.5%, PM_2.5_ = 202.5μg/m^3^, PM_10_ = 400.2μg/m^3^, Vis = 2.2 km, AQI = 450).

**Fig 3:**
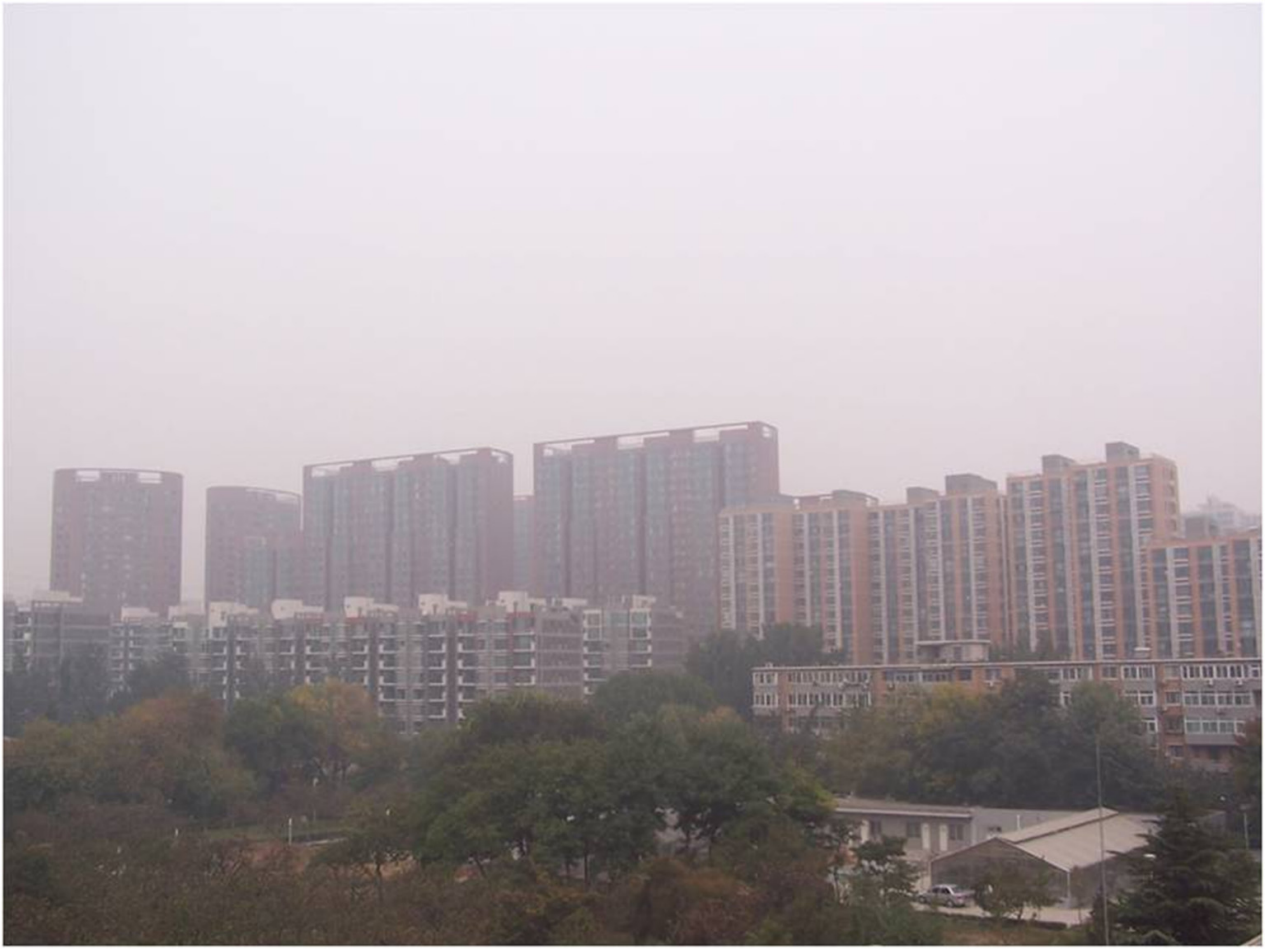
Photo No.3 in CVM (Haze Degree: Light, RH = 70.4%, PM_2.5_ = 139.6μg/m^3^, PM_10_ = 291.5μg/m^3^, Vis = 3.2 km, AQI = 402).

**Fig 4:**
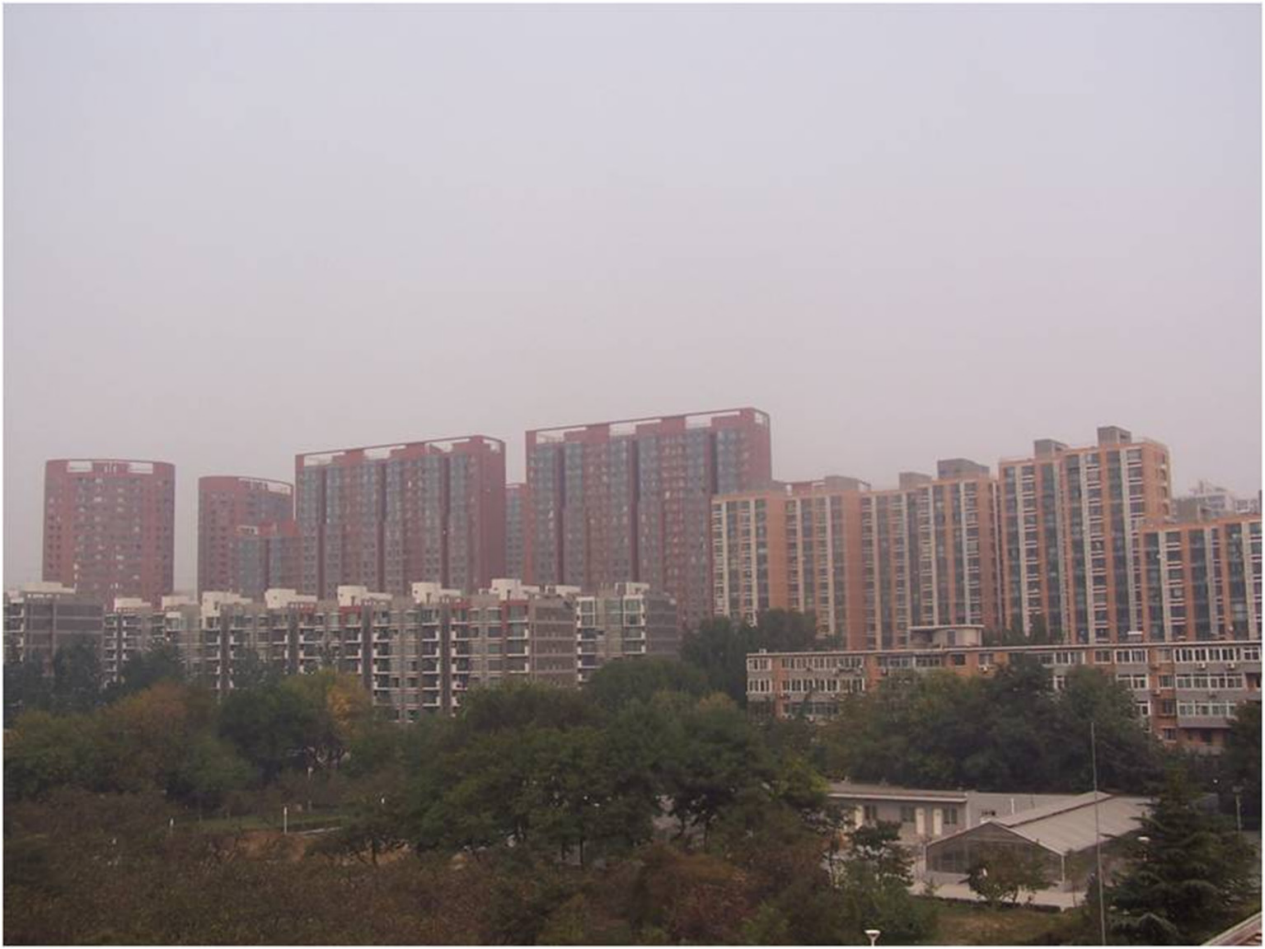
Photo No.4 in CVM (Haze Degree: Slight, RH = 64.2%, PM_2.5_ = 131.4μg/m^3^, PM_10_ = 311.7μg/m^3^, Vis = 4.9 km, AQI = 431).

**Fig 5:**
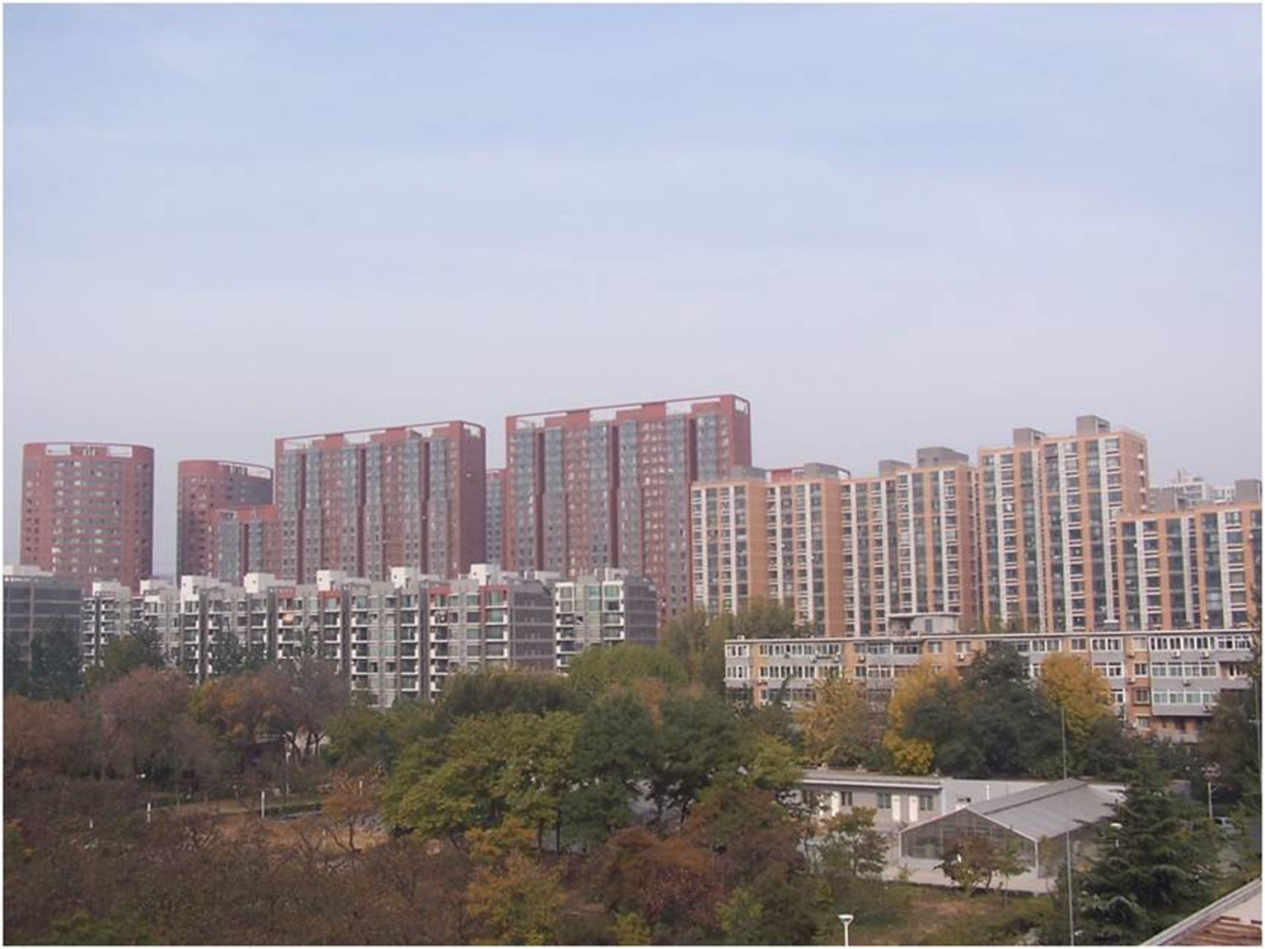
Photo No.5 in CVM (Haze Degree: Mild, RH = 54.6%, PM2.5 = 116.5μg/m^3^, PM10 = 253.9μg/m^3^, Vis = 6.9 km, AQI = 349).

There are five photos in total, which are all about heavy pollution (according to “Ambient air quality standards” GB 3095–2012) but the haze degree is from severe to slight (according to “Observation and forecasting level of haze” QX/T 113–2010). The details are shown in Figs 1–5. The mass concentrations of PM_2.5_ and PM_10_ were continuously measured using two beta attenuation monitors (Thermo FH62C14, and BAM 1020, Metone Co.), equipped with a smart heater to condition the sample air stream according to the designated reference methods of the guidelines of the USEPA. The limits of the detection of the techniques are all sufficient to accurately measure the relatively high concentration of aerosols at the study sites. The mass concentrations of PM_2.5_ and PM_10_ were amended by the filter samples, which were taken at the same site and balanced and weighted in a lab with stable temperature and humidity. The variation of temperature (T), pressure (Pre), solar radiation (SR), total ultraviolet (UV), relative humidity (RH), visibility (Vis) and wind speed (WS)/directions (WD) were measured by VISALA instruments. The upper limit of the visibility sensor was 20 km.

In particular, the place where the people live may also affect their perception and attitude toward the environment. According to the pollution characteristics and pollution level [24], city-clusters or cities was classified in this study: a) the North China Plain, where is taken as the most polluted area in China and the economy has been developed in a normal speed, including Beijing, Tianjin, Hebei province (all 11 cities), Shanxi province (all 11 cities), the north part of Shandong province (Dezhou, Liaocheng, Jinan, and Binzhou), and the south part of Mongolia (Hohehaote, Baotou, Eerduosi, and Wulanchabu); b) the Yangtze River Delta, where is less polluted than North China Plain and the economic development is quick, including Shanghai, Jiangsu province (all 13 cities), Zhejiang province (all 11 cities), the east part of Anhui province (Huaibei, Suzhou, Bengbu, Hefei, Chuzhou, Maanshan, Wuhu, Chaohu, Tongling, Xuancheng, Huangshan), and South part of Shandong (Zaozhuang, Linyi, Jining, and Heze); c) small city-clusters or cities with good air quality but poor economy, such as Kunming in Yunnan Province, Haikou in Hainan Province, and Xiamen in Fujian Province; and d) small city-clusters or cities with middle level air quality between the North China Plain and the Yangtze River Delta, such as Shandong Peninsula, Liaoning province, Sichuan province, Shanxi province. In addition, there is one special city cluster, the Pearl River Delta including all 13 cities in Guangdong province, which is quite different from the regions above as either its air quality or its economic conditions has been significantly improved in the past ten years.

To implement the empirical test, this study developed four dummy variables: Region_A, if the place is in the North China Plain it will be 1 or it will be 0; Region_B, if the place is in the Yangtze River Delta, the value will be 1 or it will be 0; Region_C, if the place is in the clean areas, the value will be 1 or it will be 0; and Region_D, if the place is in the middle level polluted cities, the value will be 1 or it will be 0. Thus, if the value of each variable is 0, the place will be in the Pearl River Delta.

### Subjective Measures

Questionnaire studies aim to describe behavior and gather people’s perceptions, opinions, attitudes and beliefs about different issues, which are widely used to describe populations or practices and establish a relationship between two or more variables [25]. Therefore, this study mainly adopts this method to measure the people’s perception of visibility, their attitude and behavior toward the environment, as well as the effect of their demographic characteristics.

#### Subjective atmospheric visibility, Willingness-to-accept (WTA) and Willingness-to-Pay (WTP)

Previous studies use CVM corresponding to environment evaluation to assess the economic value of environmental changes [26,27]. This involves using questionnaire surveys to identify whether the respondents are willing to accept or pay for a certain environment management policy or service [28].

According to Davidson et al [2001], the respondents in this study were asked to assess the atmospheric visibility and the level of their acceptance after they had seen the five photos shown to them. Two questions were asked about each photo, the first of which was “Do you think the landscape shown in the photo is highly visible?” with a five-point Likert scale from poor visibility to good visibility, while the second was “If the visibility outside is like that shown in the photo, would you like to go out?” with a five-point Likert scale from dislike to like. In other words, the former is designed to measure their perception of visibility (PV), while the latter measures their willingness to accept the current visibility (WTA in brief).

According to the previous studies [29,30], a scenario was assumed that the government is trying to adopt a pro-environment project to reduce the days when the air pollution and poor visibility occurs as shown in the photo, while the cost of this project would come from tax directly or indirectly. Then the respondents were asked how much money (per RMB in a year) they are willing to pay, if the cost would be paid from their household income. This amount of money represents the variable of willingness-to-pay (WTP in brief). To get a normal distribution, this study adopts the logarithm form of WTP as the dependent variable.

#### Pro-environmental attitude (PEA)

Environmental attitude is a psychological tendency expressed by evaluating the natural environment with some degree of favor or disfavor, and a crucial construct in the field of environmental psychology, discussed in more than half of all publications in this area [31]. Since 1960s, there have been social surveys and measurements of environmental attitude such as the early New Environmental Paradigms [32], the Environmental Concern Scale [33] and the Ecology Scale [34]. According to Milfont and Duckitt [35], environmental attitude is divided into two kinds: protection and utilization.

Many researchers support the relationship between pro-environmental attitude and pro-environmental behaviors [36,37]. However, only the cognition under a specific context determines the specific behaviors, so this study is trying to define a specific pro-environmental attitude toward the atmospheric environment. **Table 1**shows the items of this construct. A five-point Likert-scale from “1 = strongly disagree” to “5 = strongly agree” were employed. Factor loading reveals that all the items yield loadings greater than 0.5 and the Cronbach alpha of each dimension is higher than 0.6. A second-order confirmative factor analysis also shows a good fit (χ2/df =, 2.399, RMSEA = 0.046, NFI = 0.950, RFI = 0.933; IFI = 0.970, TLI = 0.960; CFI = 0.970). Thus, the revised measures fit our research context very well and the variable of PEA is the average score of the three dimensions.

**Table 1:** The results of explorative factor analysis.

Factor	Enjoyment of nature	External control	Intent of support
Item			
1.When I’m unhappy, I find it comfortable to breathe clean air in nature	0.661	—-	—-
2.Looking the mountains and blue sky is a great stress reducer for me	0.745	—-	—-
3.I get a sense of wellbeing when looking at the blue sky	0.702	—-	—-
4.I really enjoy laying on the grass and watching the birds in the sky	0.747	—-	—-
5.Industry should be required to use low-carbon pro-environmental materials	—-	0.759	—-
6.Governments should control the rate at which non-biodegradable materials are used	—-	0.788	—-
7.Controls should be placed on high pollution and high emission industry such as coal industry and chemical industry to protect the environment from pollution	—-	0.587	—-
8.If I ever get extra income I will donate some money to an environmental organization of protecting air quality	—-	—-	0.752
9.I would like to join and actively participate in an environmentalist group of protecting air quality	—-	—-	0.726
10.I would help to raise funds for environmental protection of air quality	—-	—-	0.822
11.I often try to persuade others that the atmospheric environment is an important thing	—-	—-	0.631
**Cronbach alpha**	0.760	0.655	0.760

#### Other control variables

According to Shen and Saijo [38], this study considered gender, age, household income, the number of family members, and education level, as control variables. Thus, the respondents were also asked to provide their demographic characteristics in the questionnaire.

Gender (GEN) is a dummy variable, the score of which is 1 when the respondent is male and 0 when the respondent is female.

Age (AGE) is an ordinal variable with four levels which are “18 and below”, “18 to 35”, “36–60” and “60 and above”.

Household income (INC) is also designed as an ordinal variable with four levels which are “less than 10000 RMB”, “between 10000 and 49999”, “between 50000 and 99999”, and “more than 99999”. The number of family members (NUM) is an ordinal variable with three levels: “3 or less”, “3 to 5”, and “5 and more”.

Education level (EDU) is defined as an ordinal variable from lower to higher levels which are “middle school or lower”, “high school”, “bachelor”, and “master”.

### Data Collection

The questionnaire was then translated into Chinese by professional researchers and back-translated into English by someone not involved in the research in order to allow experts to examine each survey item on both versions to establish meaning conformity [39]. Both versions were then evaluated by seven academic researchers who served as expert judges to assess face validity. According to Converse and Presser [40], if researchers have the resources to do more than one pretest, it might be best to use a participatory pretest first, then an undeclared test to check the choice of analysis and the standardization of the survey. Therefore, we also conducted two rounds of undeclared pretests for 175 college students (125 valid). During each pretest, respondents were not informed that it is a pretest and the survey was given just as we intend to conduct it in the field. Since all items used to measure each construct were derived from a thorough literature review, only minor modifications such as re-organizing the order of a few questions and also rewording headings and introductions for a better flow were suggested by the panel experts [41].

The method of dissemination was electronic, which is more convenient and efficient. The research team distributed invitations through a Chinese survey database to ask candidates to complete the questionnaire online. The research team finished three rounds of collection from July to November in 2013. In the first round, a total of 569 invitations were distributed and 220 valid questionnaires were finally collected, which yielded a valid response rate of 38.7%. In the second round, 1055 invitations were distributed and 214 valid questionnaires were returned, yielding a valid response rate of 20.3%. In the third round, 1050 invitations were distributed and 218 valid were returned, yielding a valid response rate of 20.8%. There is no significant difference of demographical distribution of the respondents in the three rounds. When combining the three sets of data together, we finally obtained 652 valid questionnaires with a response rate of 24.4%. As each respondent was asked to assess five photos, the total number of records was five times the original sample, which accounted for 3260 pieces of data. The sample frame is shown in **Table 2**.

**Table 2:** Sample frame.

	Valid	N	%
**Gender**	Female	373	57.2
	Males	279	42.8
**Age**	18–35	554	85.0
	36–60	98	15.0
**Household Income (RMB)**	9999 or less	19	2.9
	10000–49999	153	23.5
	50000–99999	250	36.8
	100000 or more	240	36.8
**Number of Families**	Less than 3	102	15.6
	3–5	523	80.0
	More than 5	27	4.1
**Education**	A middle school degree or below	1	0.2
	A high school degree	32	4.9
	A Bachelor’s degree	542	83.1
	A Master’s degree or above	77	11.8
**Location**	North China Plain	254	39.0
	Yangtze River Delta	180	27.6
	Pearl River Delta	50	7.6
	The middle level of polluted cities	77	11.8
	The clean cities	91	13.9

**Fig 6:**
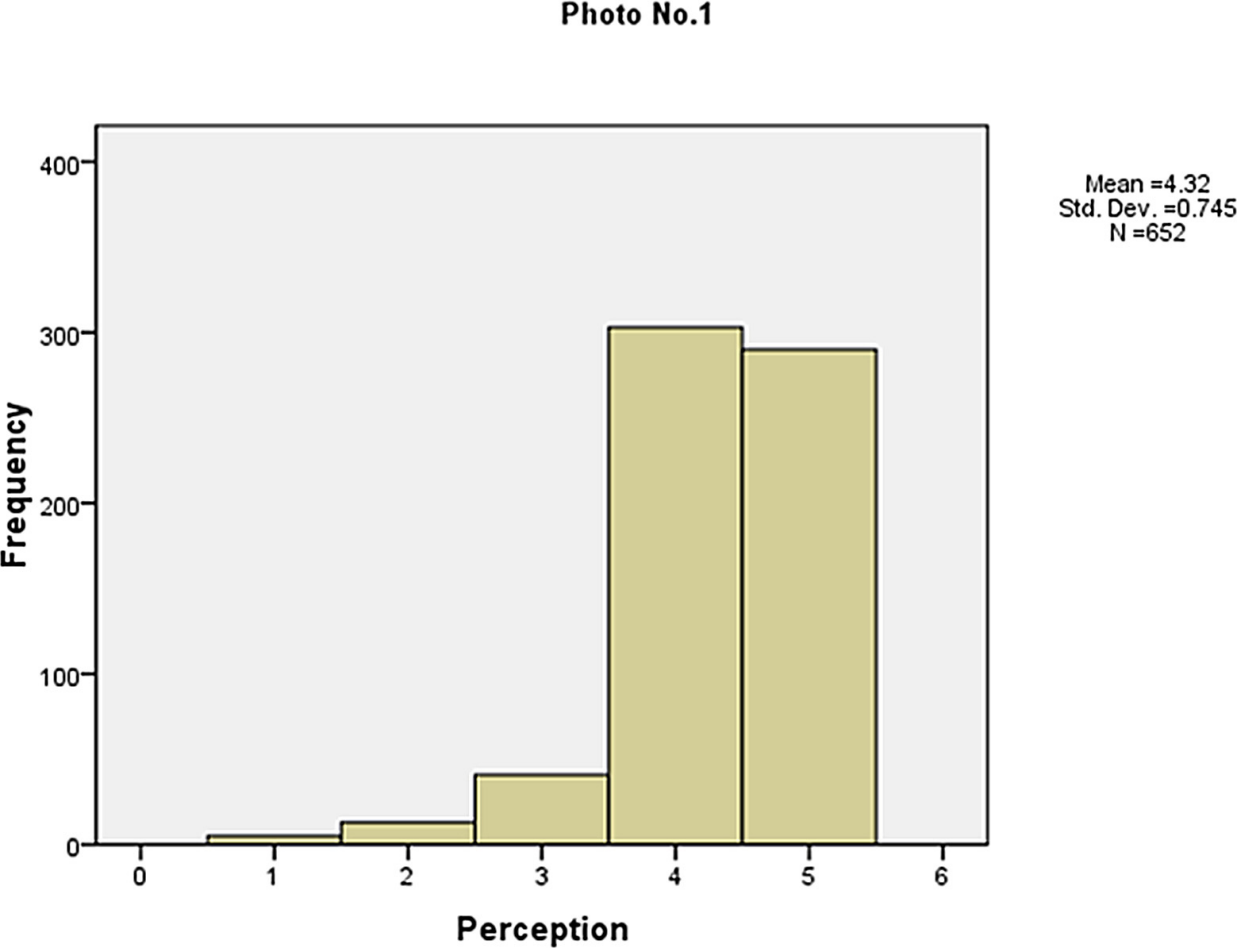
The frequency of the visibility perception (Photo No.1).

**Fig 7:**
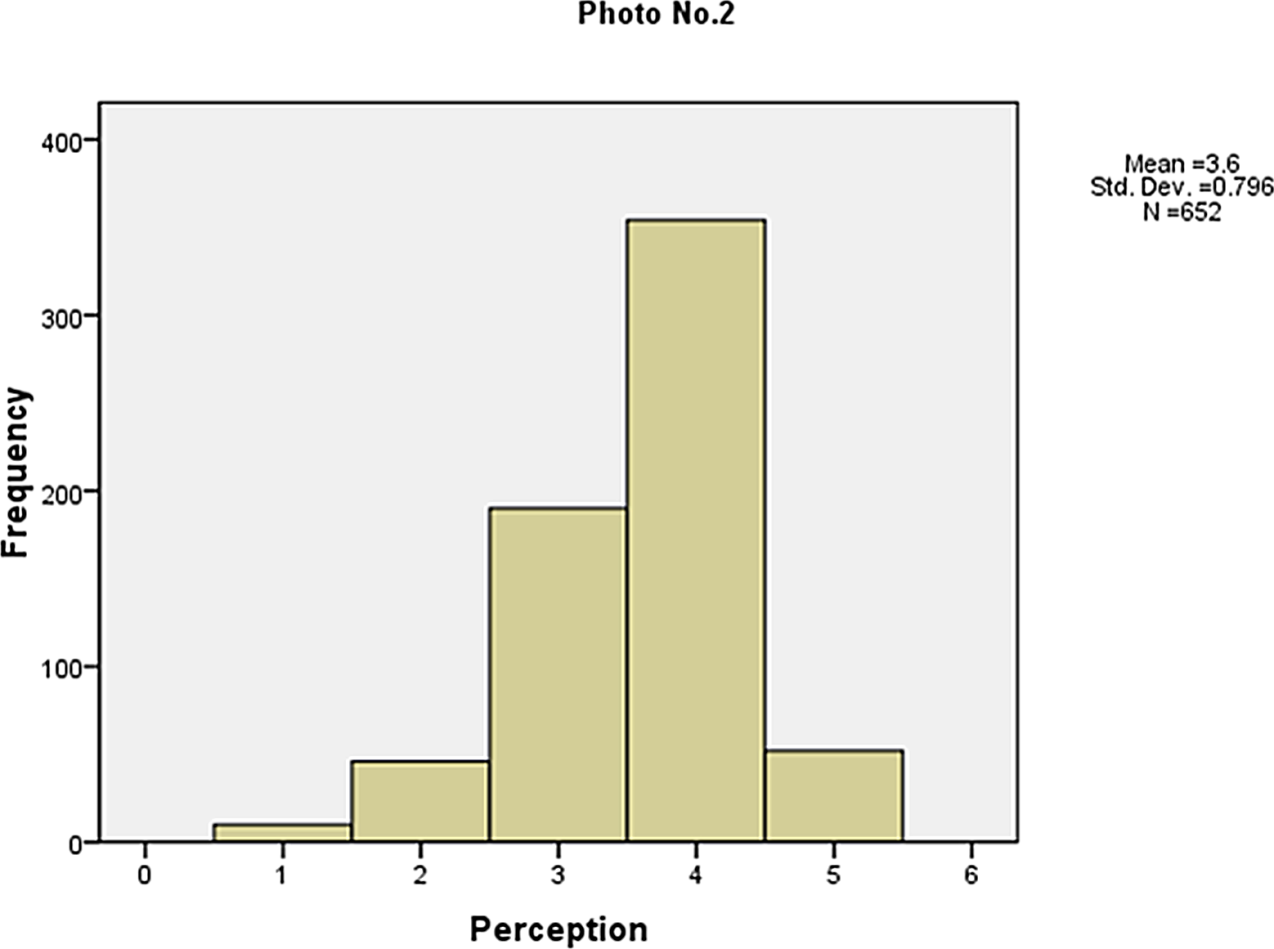
The frequency of the visibility perception (Photo No.2).

**Fig 8:**
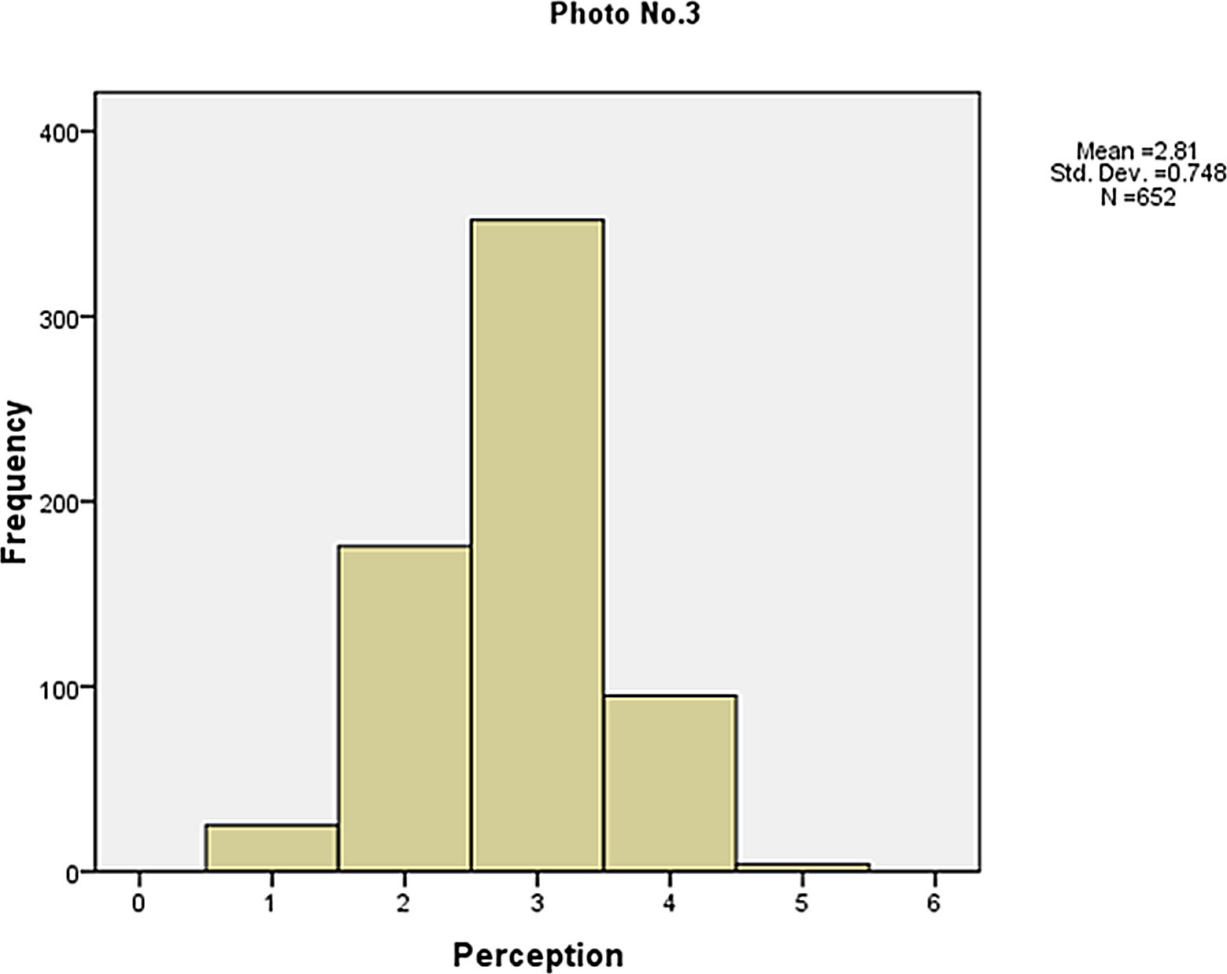
The frequency of the visibility perception (Photo No.3).

**Fig 9:**
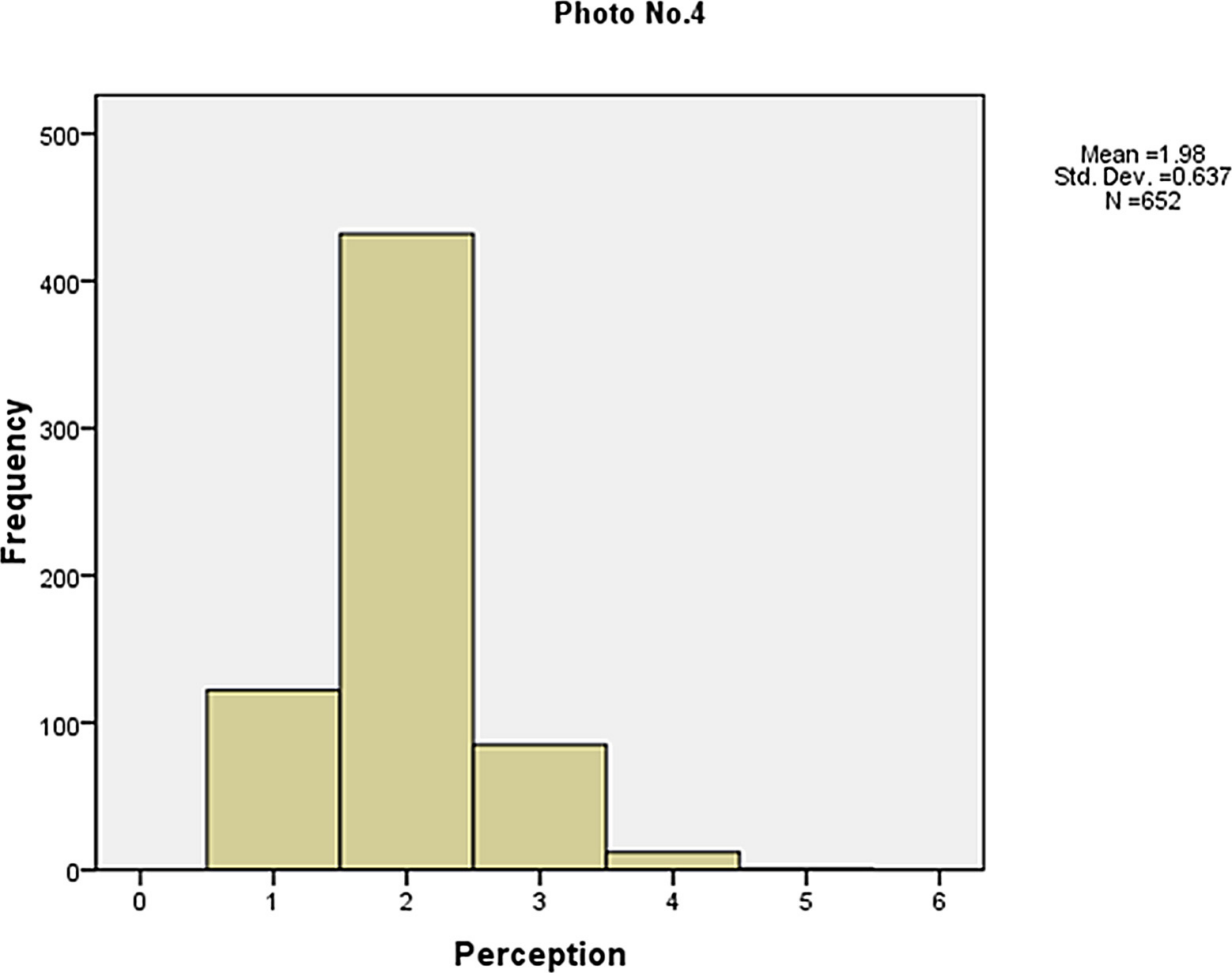
The frequency of the visibility perception (Photo No.4).

**Fig 10:**
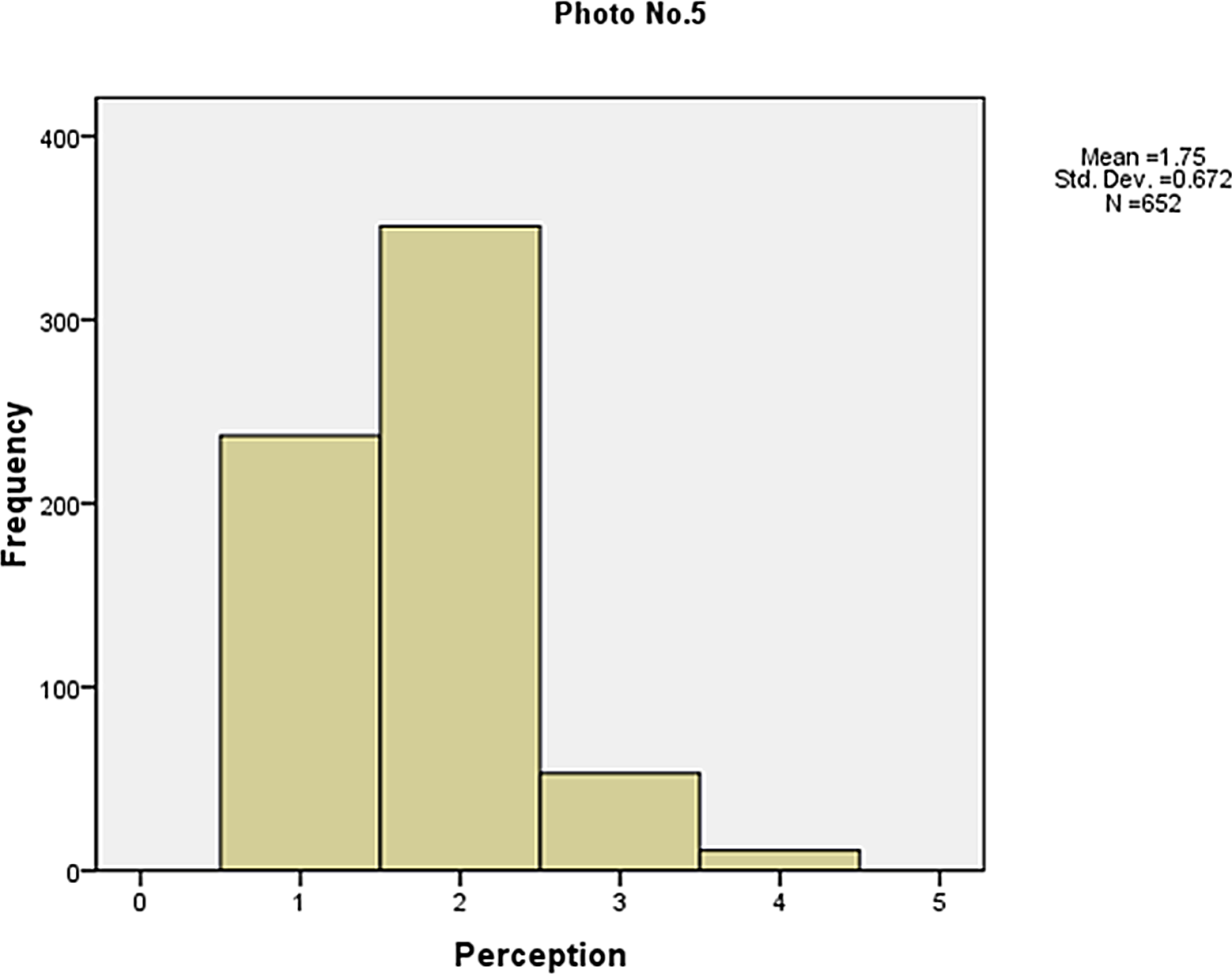
The frequency of the visibility perception (Photo No.5).

The frequency of the visibility perception is shown in Figs 6–10. Not as simple as the objective measures of visibility, the respondents assessed the same photos differently, although most of them made a correct judgement. The difference between the subjective and objective levels demonstrates the necessity to explore the mechanisms behind.

## Results

We adopted regressions to test the relationship among the key variables using the software STATA 10.0. In addition, to test the mediating effect of subjective atmospheric visibility representing the generative mechanism through which objective measures influence WTA/WTP, we adopted the well-known procedure for determining mediation presented by Baron and Kenny [42]. The results are presented in the next sections.

### The effects on WTA

If the dependent variable is not simply segmented into high WTA and low WTA but into ordered multiple categories including five levels from dislike to like, there will be four Logit regression models, which is a Cumulative Logit Model (CLM). Since the WTA in this study is from level 1 to level 5, the corresponding probabilities are p_WTA1_, p_WTA2_, p_WTA3_, p_WTA4_, p_WTA5_ and the models fitted are as follows:

$$\text{logit}\left(\frac{{\text{p}}_{\text{WTA}1}}{1-{\text{p}}_{\text{WTA}1}}\right)=\text{logit}\left(\frac{{\text{p}}_{\text{WTA}1}}{{\text{p}}_{\text{WTA}2}+{\text{p}}_{\text{WTA}3}+{\text{p}}_{\text{WTA}4}+{\text{p}}_{\text{WTA}5}}\right)=-\alpha_{1}+\beta_{1-1}\text{RH}+\beta_{1-2}\text{PM}2.5+\beta_{1-3}\text{Vis}+\beta_{1-4}\text{MALE}+\beta_{1-5}\text{AGE}+\beta_{1-6}\text{INC}+\beta_{1-7}\text{NUM}+\beta_{1-8}\text{EDU}+\beta_{1-9}{\text{Region}}_{\text{A}}+\beta_{1-10}{\text{Region}}_{\text{B}}+\beta_{1-11}{\text{Region}}_{\text{C}}+\beta_{1-12}{\text{Region}}_{\text{D}}$$ (1)

$$\text{logit}\left(\frac{{\text{p}}_{\text{WTA}1}+{\text{p}}_{\text{WTA}2}}{1-\left({\text{p}}_{\text{WTA}1}+{\text{p}}_{\text{WTA}2}\right)}\right)=\text{logit}\left(\frac{{\text{p}}_{\text{WTA}1}+{\text{p}}_{\text{WTA}2}}{{\text{p}}_{\text{WTA}3}+{\text{p}}_{\text{WTA}4}+{\text{p}}_{\text{WTA}5}}\right)=-\alpha_{2}+\beta_{2-1}\text{RH}+\beta_{2-2}\text{PM}2.5+\beta_{2-3}\text{Vis}+\beta_{2-4}\text{MALE}+\beta_{2-5}\text{AGE}+\beta_{2-6}\text{INC}+\beta_{2-7}\text{NUM}+\beta_{2-8}\text{EDU}+\beta_{2-9}{\text{Region}}_{\text{A}}+\beta_{2-10}{\text{Region}}_{\text{B}}+\beta_{2-11}{\text{Region}}_{\text{C}}+\beta_{2-12}{\text{Region}}_{\text{D}}$$ (2)

$$\text{logit}\left(\frac{{\text{p}}_{\text{WTA}1}+{\text{p}}_{\text{WTA}2}+{\text{p}}_{\text{WTA}3}}{1-\left({\text{p}}_{\text{WTA}1}+{\text{p}}_{\text{WTA}2}+{\text{p}}_{\text{WTA}3}\right)}\right)=\text{logit}\left(\frac{{\text{p}}_{\text{WTA}1}+{\text{p}}_{\text{WTA}2}+{\text{p}}_{\text{WTA}3}}{{\text{p}}_{\text{WTA}4}+{\text{p}}_{\text{WTA}5}}\right)=-\alpha_{3}+\beta_{3-1}\text{RH}+\beta_{3-2}\text{PM}2.5+\beta_{3-3}\text{Vis}+\beta_{3-4}\text{MALE}+\beta_{3-5}\text{AGE}+\beta_{3-6}\text{INC}+\beta_{3-7}\text{NUM}+\beta_{3-8}\text{EDU}+\beta_{3-9}{\text{Region}}_{\text{A}}+\beta_{3-10}{\text{Region}}_{\text{B}}+\beta_{3-11}{\text{Region}}_{\text{C}}+\beta_{3-12}{\text{Region}}_{\text{D}}$$ (3)

$$\text{logit}\left(\frac{{\text{p}}_{\text{WTA}1}+{\text{p}}_{\text{WTA}2}+{\text{p}}_{\text{WTA}3}+{\text{p}}_{\text{WTA}4}}{1-\left({\text{p}}_{\text{WTA}1}+{\text{p}}_{\text{WTA}2}+{\text{p}}_{\text{WTA}3}+{\text{p}}_{\text{WTA}4}\right)}\right)=\text{logit}\left(\frac{{\text{p}}_{\text{WTA}1}+{\text{p}}_{\text{WTA}2}+{\text{p}}_{\text{WTA}3}+{\text{p}}_{\text{WTA}4}}{{\text{p}}_{\text{WTA}5}}\right)=-\alpha_{4}+\beta_{4-1}\text{RH}+\beta_{4-2}\text{PM}2.5+\beta_{4-3}\text{Vis}+\beta_{4-4}\text{MALE}+\beta_{4-5}\text{AGE}+\beta_{4-6}\text{INC}+\beta_{4-7}\text{NUM}+\beta_{4-8}\text{EDU}+\beta_{4-9}{\text{Region}}_{\text{A}}+\beta_{4-10}{\text{Region}}_{\text{B}}+\beta_{4-11}{\text{Region}}_{\text{C}}+\beta_{4-12}{\text{Region}}_{\text{D}}$$ (4)

Where RH is the relative humidity; PM_2.5_ represents the mass concentration of particles smaller than 2.5μm; Vis is the objective atmospheric visibility shown in the photos; MALE is a dummy variable (male is coded as 0, and female is coded as 1); AGE stands for the respondents’ age; INC for the respondents’ household income (RMB per year); NUM for the number in the family; EDU is a dummy variable representing the respondents’ educational level (Bachelor’s degree is coded as 1, Master’s degree is coded as 0); Region_A, Region_B, Region_C and Region_D are all dummy variable representing the permanent residence of the respondents (1, 0, 0, 0) for the North China Plain, (0, 1, 0, 0) for the Yangtze River Delta, (0, 0, 1, 0) for the clean cities and (0, 0, 0, 1) for the middle level polluted places and (0, 0, 0, 0) for the Pearl River Delta.

Only the functions of the first Logit regression are given for the next two models, as follows, and the other Logit regressions are easy to establish similar to functions (2) to (4).

$$\text{logit}\left(\frac{{\text{p}}_{\text{WTA}1}}{1-{\text{p}}_{\text{WTA}1}}\right)=\text{logit}\left(\frac{{\text{p}}_{\text{WTA}1}}{{\text{p}}_{\text{WTA}2}+{\text{p}}_{\text{WTA}3}+{\text{p}}_{\text{WTA}4}+{\text{p}}_{\text{WTA}5}}\right)=-\alpha_{5}+\beta_{5-1}\text{RH}+\beta_{5-2}\text{PM}2.5+\beta_{5-3}\text{VIS}+\beta_{5-4}\text{MALE}+\beta_{5-5}\text{AGE}+\beta_{5-6}\text{INC}+\beta_{5-7}\text{NUM}+\beta_{5-8}\text{EDU}+\beta_{5-9}{\text{Region}}_{\text{A}}+\beta_{5-10}{\text{Region}}_{\text{B}}+\beta_{5-11}{\text{Region}}_{\text{C}}+\beta_{5-12}{\text{Region}}_{\text{D}}+\beta_{5-12}\text{PV}$$ (5)

$$\text{logit}\left(\frac{{\text{p}}_{\text{VIS}1}}{1-{\text{p}}_{\text{PV}1}}\right)=\text{logit}\left(\frac{{\text{p}}_{\text{VIS}1}}{{\text{p}}_{\text{PV}2}+{\text{p}}_{\text{PV}3}+{\text{p}}_{\text{PV}4}+{\text{p}}_{\text{PV}5}}\right)=-\alpha_{6}+\beta_{6-1}\text{RH}+\beta_{6-2}\text{PM}2.5+\beta_{6-3}\text{VIS}+\beta_{6-4}\text{MALE}+\beta_{6-5}\text{AGE}+\beta_{6-6}\text{INC}+\beta_{6-7}\text{NUM}+\beta_{6-8}\text{EDU}+\beta_{6-9}{\text{Region}}_{\text{A}}+\beta_{6-10}{\text{Region}}_{\text{B}}+\beta_{6-11}{\text{Region}}_{\text{C}}+\beta_{6-12}{\text{Region}}_{\text{D}}$$ (6)

Where PV is the visibility level perceived by the respondents (Likert–5)

As shown in **Table 3**, and Model 1 tests the effect of objective visibility measures, including RH, PM_2.5_ and Vis, on PV; Model 2 tests the effect of objective visibility measures, including RH, PM_2.5_ and Vis, on WTA when controlling the respondents’ demographic characteristics; and Model 3 tests the effect of measure PV on WTA when controlling objective visibility measures and the respondents’ demographic characteristics. The results illustrate that RH and PM_2.5_ both have a significant negative effect on WTA (*β* = -0.084, *p*<0.001; *β* = -0.026, *p*<0.001), while Vis has a significant positive effect on WTA (*β* = 0.421, *p*<0.001). However, when the subjective measure of visibility was entered together with the objective measures, these effects became smaller but still significant, while the effect of PV was positive and significant (*β* = 2.320, *p*<0.001). As the effects of RH, PM_2.5_ and Vis on PV are all significant (*β* = -0.092, *p*<0.001; *β* = -0.029, *p*<0.001; *β* = 0.536, *p*<0.001), the mediating effect of PV is supported. Besides, Region_A, Region B and Region_C all have significant positive effect on PV (*β* = 0.439, *p*<0.05; *β* = 0.504, *p*<0.01; *β* = 0.489, *p*<0.05), while only Region_B has a significant positive effect on WTA (*β* = 0.045, *p*<0.05). In addition, the control variables such as AGE has significant and negative effects on both PV and WTA, while INC has a positive effect on PV but a negative effect on WTA.

**Table 3:** The results of regressions.

DV	$\text{logit}\left(\frac{{\text{p}}_{\text{PV}1}}{1-{\text{p}}_{\text{PV}1}}\right)$	$\text{logit}\left(\frac{{\text{p}}_{\text{WTA}1}}{1-{\text{p}}_{\text{WTA}1}}\right)$	$\text{logit}\left(\frac{{\text{p}}_{\text{WTA}1}}{1-{\text{p}}_{\text{WTA}1}}\right)$	Ln(WTP)
IV	Model 1	Model 2	Model 3	Model 4
Objective measures				
RH	-0.092^***^	-0.084^***^	-0.034^***^	0.004^+^
	0.012	0.010	0.012	0.002
PM_2.5_	-0.029^***^	-0.026^***^	-0.009^***^	0.001^+^
	0.002	0.002	0.003	0.001
Vis	0.536^***^	0.421^***^	0.160^*^	-0.029^*^
	0.079	0.069	0.079	0.014
Subjective measure				
PV	—-	—-	2.320^***^	0.127^*^
			0.109	0.065
PEA	—-	—-	—-	0.588^***^
				0.174
Demographic characters				
MALE	-0.015	0.126	0.183	-0.040
	0.115	0.112	0.123	0.116
AGE	-0.439^**^	-0.455^**^	-0.224	-0.368^*^
	0.176	0.172	0.174	0.176
INC	0.177^**^	0.089	-0.034^*^	0.217^**^
	0.071	0.067	0.065	0.075
NUM	0.144	0.087	-0.219	0.084
	0.148	0.132	0.141	0.139
EDU	0.089	0.059	-0.123	0.316^*^
	0.152	0.129	0.153	0.143
Region_A	0.439^*^	0.388^*^	0.144	-0.300
	0.192	0.198	0.204	0.214
Region_B	0.504^**^	0.347^+^	0.045^*^	-0.475^*^
	0.203	0.204	0.207	0.217
Region_C	0.489^*^	0.320^*^	0.004	-0.329
	0.224	0.232	0.237	0.249
Region_D	0.201	0.121	0.002	-0.403^+^
	0.242	0.272	0.272	0.242
_con1	-11.845	-11.413	-1.134	1.709
	1.656	1.488	1.680	1.124
_con2	-8.989	-9.447	1.482	—-
	1.594	1.451	1.649	
_con3	-6.846	-7.565	4.270	—-
	1.586	1.441	1.653	
_con4	-3.953	-5.144	7.661	—-
	1.611	1.455	1.687	
n	3260	3260	3260	3175
χ2/F	852.63^***^	914.26^***^	984.83^***^	3.59^***^
R2	32.36%	27.09%	43.17%	7.2%

Note: The first line in each cell is the coefficient, while the second line is the standard error.

+p<0.1

*p<0.05

**p<0.01

***p<0.001.

**Fig 11:**
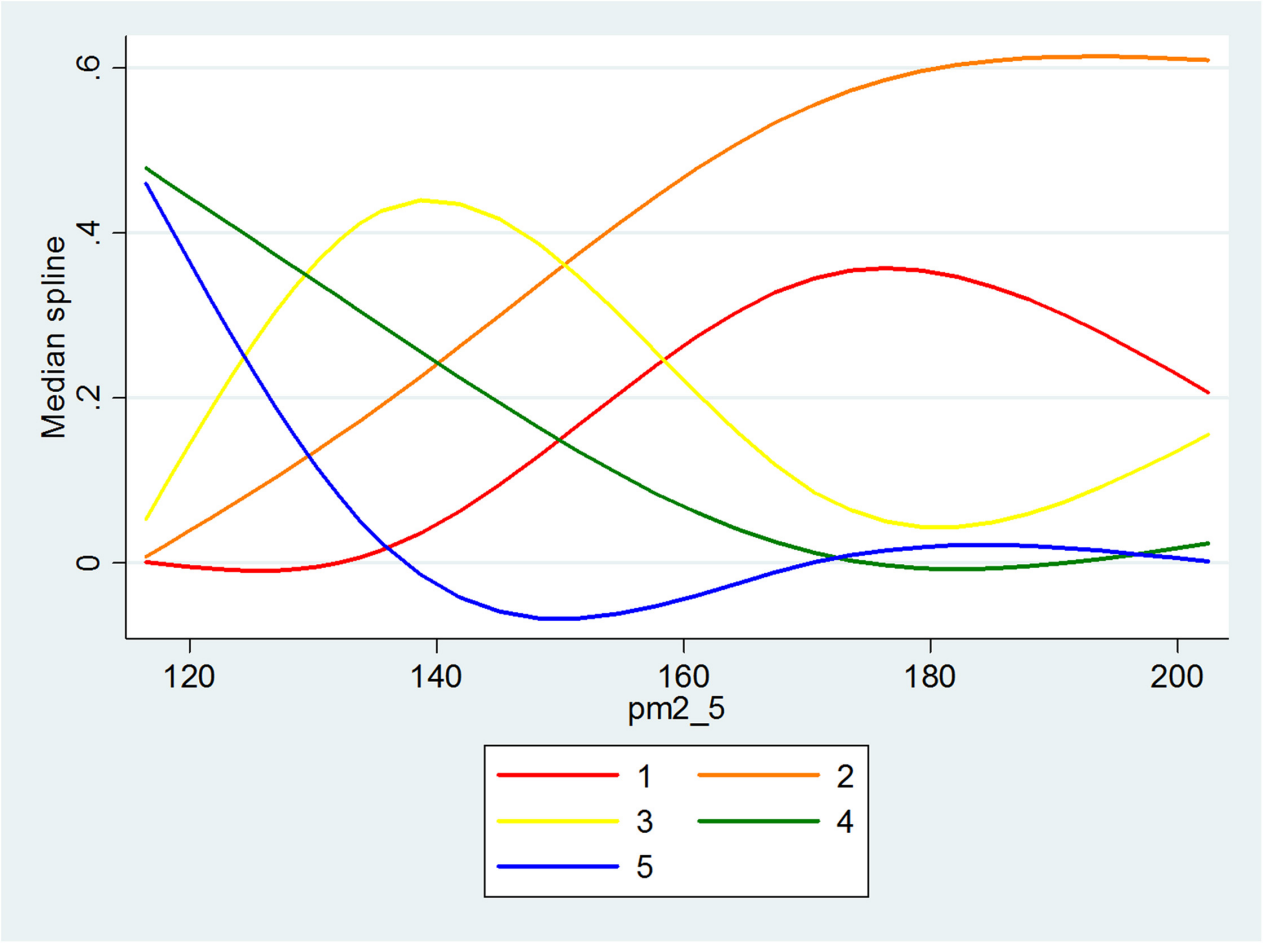
The relationship between Vis and PV.

**Fig 12:**
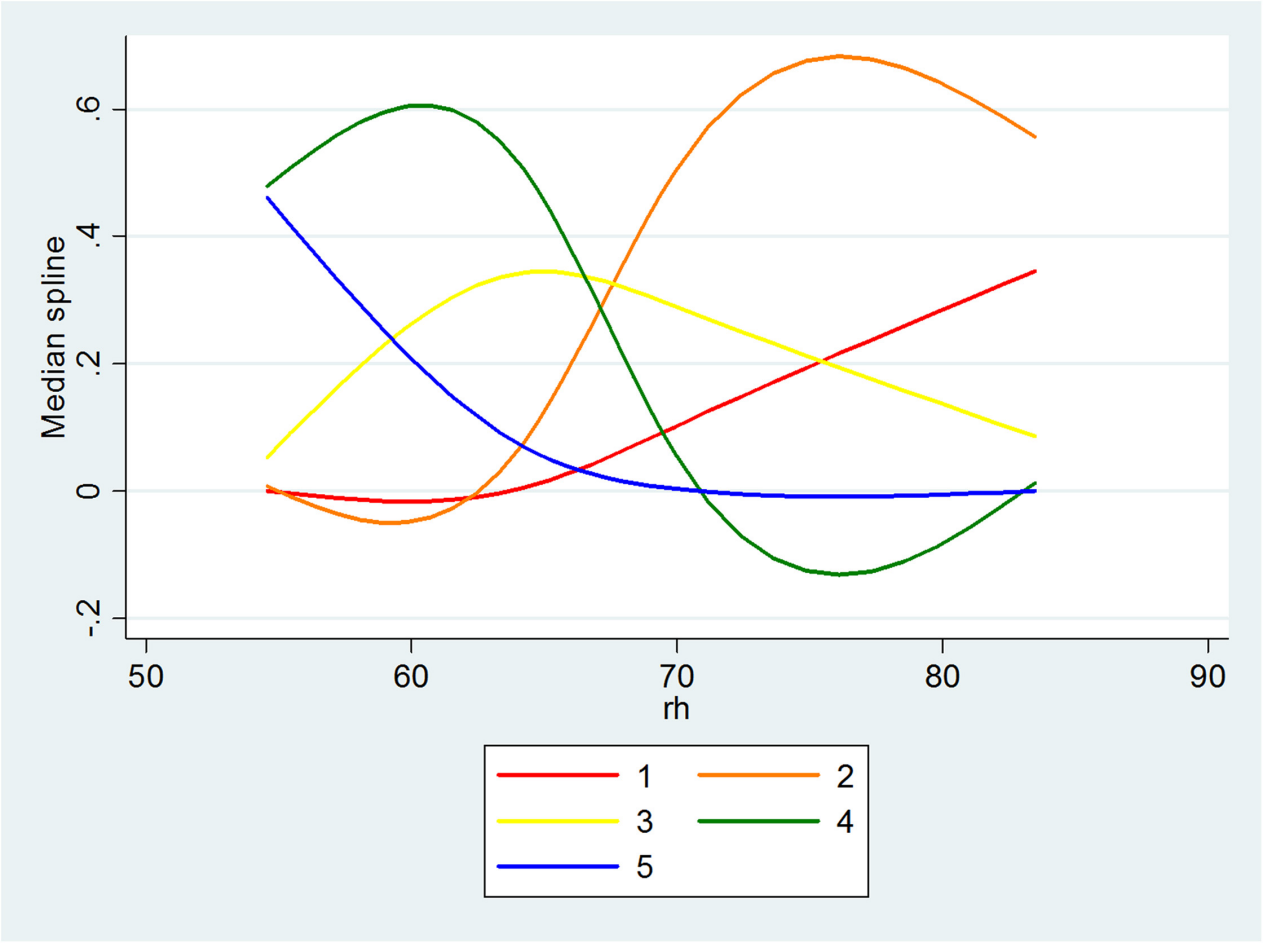
The relationship between RH and PV.

**Fig 13:**
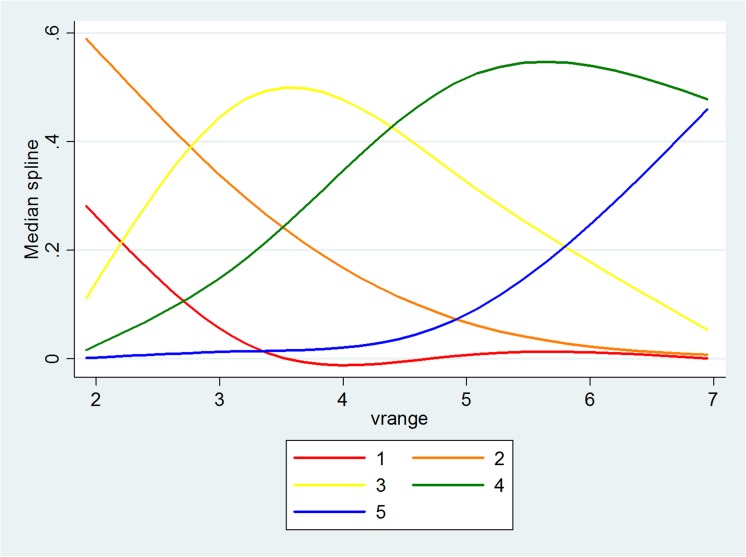
The relationship between PM2.5 and PV.

**Fig 14:**
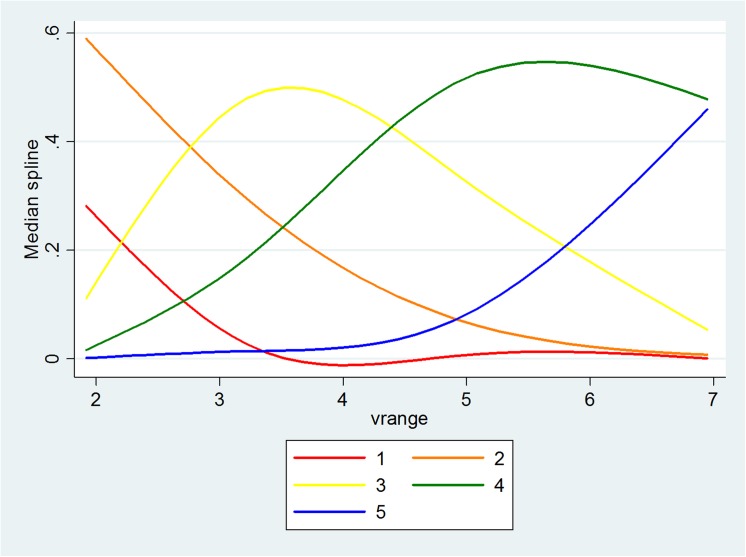
The relationship between PV and WTA.

The significant key relationships in Model 1 are described in Figs 11–13, with the vertical axis representing the probability of five levels of PV and the horizontal axis representing the objective measure of visibility. When the level of RH increases to a value of around 70, the distance between the probability of the lower level of PV and the probability of the higher level of PV increases. Also, when the level of PM_2.5_ increases to a value of around 160μg/m^3^, the probabilities also become distinctive. Although this is not significant for the relationship between Vis and PV, it seems that a value of Vis of around 5 is the turning point. Fig 14 illustrates the relationship in Model 3 with the vertical axis representing the probability that there are five levels of WTA and the horizontal axis represents PV. The red line to the blue line stands for the lowest level of WTA to highest level of WTA; therefore, as PV increases, the probability of the lowest level of WTA drops quickly and the probability of the highest level of WTA rises quickly.

### The effects on WTP

As explained earlier, willingness-to-pay is measured as a continuous variable and the logarithm form is taken to keep normal distribution. Thus, in Function (7), ln(WTP) is the dependent variable, while the objective measures (RH, PM_2.5_, and Vis) and the subjective measure- perception of visibility (PV) are independent variable, controlling the variables of demographic characters and pro-environmental attitude (PEA) as it is emphasized as a key factor affecting environmental behaviors in environmental psychological studies.

$$\text{ln}\left(\text{WTP}\right)=\alpha_{7}+\beta_{7-1}\text{RH}+\beta_{7-2}\text{PM}2.5+\beta_{7-3}\text{Vis}+\beta_{7-4}\text{MALE}+\beta_{7-5}\text{AGE}+\beta_{7-6}\text{INC}+\beta_{7-7}\text{NUM}+\beta_{7-8}\text{EDU}+\beta_{7-9}{\text{Region}}_{\text{A}}+\beta_{7-10}{\text{Region}}_{\text{B}}+\beta_{7-11}{\text{Region}}_{\text{C}}+\beta_{7-12}{\text{Region}}_{\text{D}}+\beta_{7-13}\text{PV}+\beta_{7-14}\text{PEA}$$ (7)

Where PEA is the level of pro-environmental attitude of the respondents (Likert–5)

As Shown in Table 3, Model 4 is testing the effects on willingness-to-pay. The results show that among the objective measures, only objective atmospheric visibility has a significant but negative effect on WTP (β = -0.029, p<0.05). Also, the effect of subjective atmospheric visibility is significant and positive on WTP (β = -0.029, p<0.05), but it is much smaller than the effect on WTA (β = 0.127, p<0.05). In particular, the effect of pro-environmental attitude is positive and very significant (β = 0.588, p<0.001). Furthermore, Region B has a significant but negative effect on WTP (*β* = -0.475, *p*<0.05), while Region_D only has a marginal and positive effect on WTP (*β* = -0.403, *p*<0.1). In addition, the control variable AGE has a significant and negative effect on WTP, and obviously INC has a significant and positive effect on WTP.

## Discussion

Both objective measures and subjective measures of visibility are important in designing the urban landscape. The former, which consists of physical “hard” measures observed by technological instruments, illustrates how well environmental characteristics meet the criteria that was believed to be necessary for a good life, while the latter based on “soft” psychological responses, such as perceptions, enables a study of the way in which people assess whether or not environmental characteristics satisfy their needs. The results show that the people’s perception of visibility is also based on objective information about the ambient air (RH, PM_2.5_ and Visibility), which means that one part of the people’s judgment comes from knowledge generated from the information collected and processed in society, while the other part may be based on certain psychological factors. The people’s pro-environmental behavior could also be guided in the right direction with the distribution of more accurate information. It is also found that there are some turning points that could enable people to distinguish good and bad air quality. The shape of the curves offers the points, namely, after RH = 70%, PM_2.5_ = 160μg/m^3^, Visibility = 5km, the respondents had a clear perception of the visibility shown in the photos being either good or poor.

**Fig 15:**
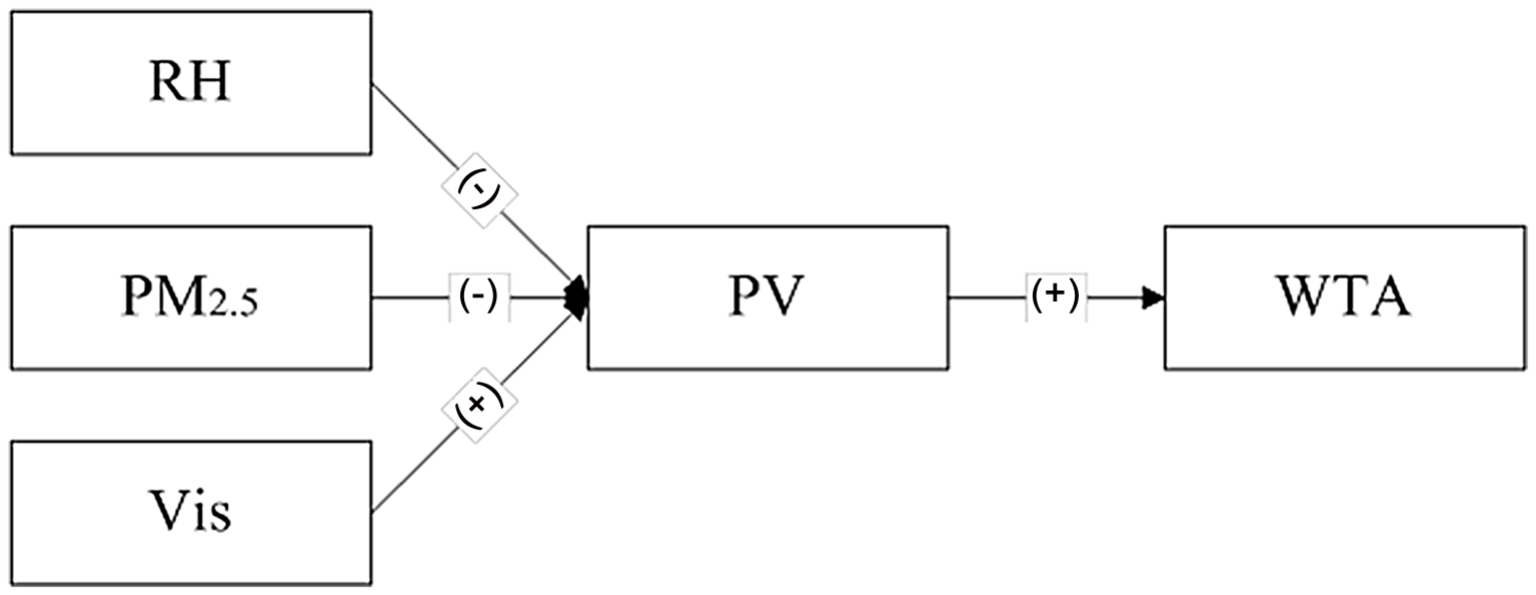
The tested mediating effects for WTA.

**Fig 16:**
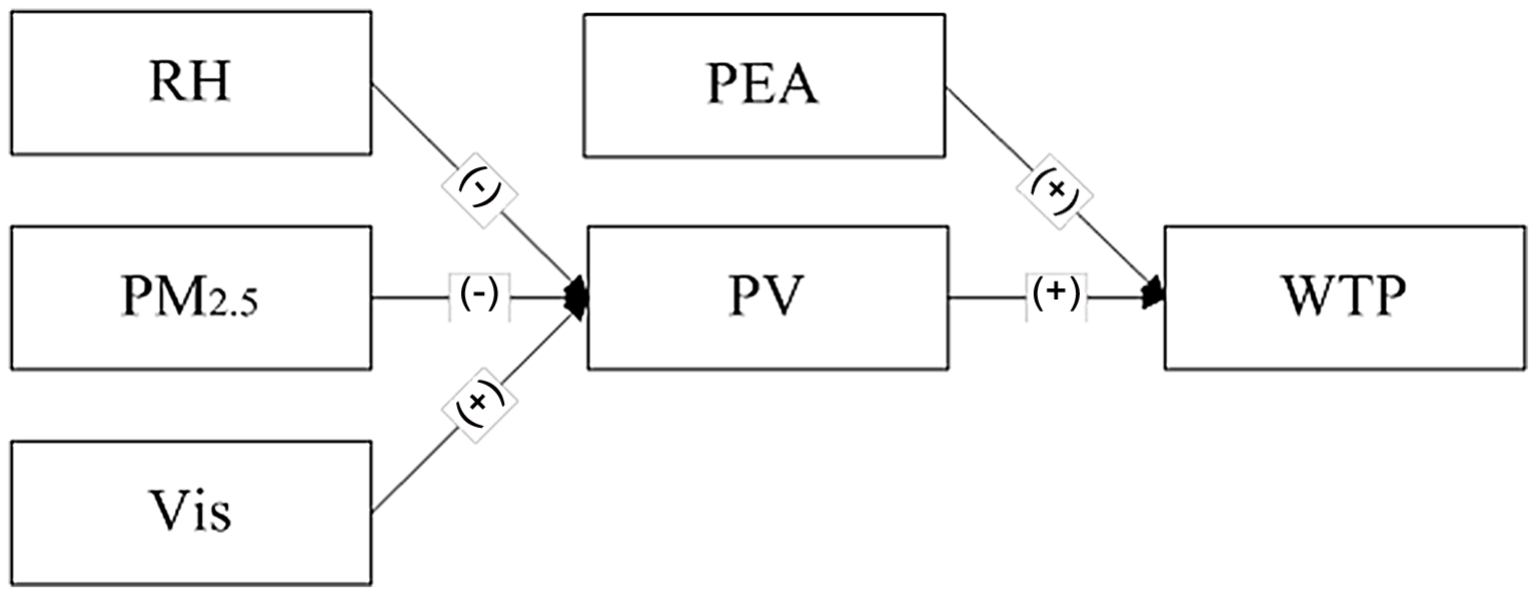
The tested mediating effects for WTP.

The results based on regressions support the fact that the subjective measure of visibility mediates the effect of the objective measure of visibility on willingness-to-accept or willingness-to-pay. Figs 15 and 16 show the results based on regressions that subjective atmospheric visibility mediates the effect of all three objective indicators. The lower the level of RH and PM_2.5_ as well as the higher level of visibility shown in the photo, the more respondents would assess a higher level of atmospheric visibility, and the more respondents would illustrate a higher level of willingness to accept this condition or pay for improving the air quality. It is easy to understand that a higher perception of visibility leads to the greater intention to go outside, which reflects the willingness to accept. However, pushing people to pay for the improvement of visibility is not consistent with the normal awareness. This suggests that under the same condition, if the people perceive the ambient visibility is much better than the other people do, they would like to more strongly support the pro-environmental policies. In other words, the over-fit between subjective and objective atmospheric visibility indicates a positive affection, which stimulates more invests in getting a better feedback. Therefore, underlying the effect of subjective atmospheric visibility on WTA, there may be another mediator of positive affection, which would be investigated in the further study.

The main difference between the two figures is that pro-environmental attitude has a much more significant effect on willingness-to-pay than subjective atmospheric visibility does, which is consistent with the existing studies. It means that the higher the level of pro-environmental attitude is and the higher the level of subjective atmospheric visibility, the more people will pay to improve the atmospheric visibility, but the former effect is much stronger. This is because the underlying mechanisms are different that one is the power of attitude and the other is from the view of perception which is based on the objective attributes of the environment. In China, the public has not paid more attention on visibility than before, after smoggy weather affected a total area of 1.43 million square kilometers and a population of 800 million people at the start of 2013 [43]. In fact, in a long time, the Chinese people have overlooked the influence of the poor air quality and they just begin to aware the tense situation according to more and more information about air pollutions exposed by the media. However, it will take a long time for them to realize the importance of taking conscious actions to protect the atmospheric environment. Thus, the results of this study also enlighten the government that it is essential to raise people’s awareness of the subjective measures of visibility, and more important, cultivate their pro-environmental attitude to gain further pro-environmental support.

Furthermore, it is found that the respondents from North China Plain, Yangtze River Delta or the clean areas have higher level of subjective atmospheric visibility. In other words, the people living in the most heavily polluted regions or the cleanest regions intend to have higher level of visibility perception than the others. The reasons may be that the respondents from North China Plain and Yangtze River Delta where the polluted issue is quite serious are sensitive to the low level of visibility, so their perception of visibility would rise to a much higher level even when the objective level of pollution decreases a little bit. While the respondents from the unpolluted areas, they may not be sensitive to the air quality because of the small variance and they may have less experience to distinguish a bad condition and a worse one, so their perception of visibility would still be higher than the others even under a low level of air quality. With an increasing number of air pollution episodes and low visibility days reported by the media from the end of 2010, the Chinese government has begun to take more actions to reducing air pollutant emissions and improving air quality across cities, municipalities, and provinces. However, it is very difficult to make a general plan for different regions in China. The findings of this study could also help the local government to implement corresponding policies targeting at the people in the specific region. For example, the promotion of environmental protection would arouse the people in the most heavily polluted or the cleanest regions, while this may not be significant in other places.

## Limitations

This study has some limitations, the first of which is that respondents were only given five photos to distinguish the visibility. Although every attempt was made to diversify the degree of air quality shown in these photos, it would have been better to show more photos randomly. Future studies could design a number of experiments in which the respondents are asked to answer the questions straightly after showing them a random group of photos. Secondly, these photos are taken in natural conditions, but there is a method whereby the landscape can be simulated by combining the objective indicators. Future studies could try to use certain software to achieve this and compare the results with the real photos. Thirdly, although the measure of willingness-to-pay is direct and convenient, it is quite difficult for the respondents to give the accurate amount of payment and the amounts estimated by heterogonous populations are often dispersed in a wide range. Thus, future studies could try many other different methods, such as biding experiments [44] and choice modeling [45] etc. Finally, only some basic demographic characteristics of the respondents were controlled in the models, whereas more complex psychological factors, such as their value orientation, affection and so on could also affect their behavioral intention, which could be considered in future studies.

## Conclusions

Many studies of visibility only focus on objective measures while neglecting the importance of subjective ones. However, the quality of the urban environment may be investigated from the perspective of either an “expert” or a “lay person”, so this study distinguished the objective measures from the subjective measures of visibility. Based on both a lab experiment and a field survey, data was collected to support the framework that the people’s perception of visibility mediates the effect of objective measures of visibility on their willingness to accept the air quality or pay for the improvement. Therefore, attention to the subjective atmospheric visibility is critical to gain the pro-environmental support of a people whose behavioral intentions would be guided by more accurate information about the air quality. Also, the environment-protection promoting policy, public participation or air pollution control strategy should be diversified in different regions of China, since the residential context could also affect people’s judgment.

## References

[1] Chan CK, Yao X. Air pollution in mega cities in China. Atmospheric Environment 2008; 42: 1–42.

[2] Watson J G. Visibility: Science and Regulation. Journal of the Air and Waste Management Association 2002; 52(6): 628–713. 12074426 10.1080/10473289.2002.10470813

[3] Bell ML, Dominici F, Ebisu K, Zeger SL, Samet JM. Spatial and temporal variation in PM2.5 chemical composition in the United States for health effects studies. Environmental Health Perspectives 2007; 115: 989–995. 17637911 10.1289/ehp.9621 PMC1913582

[4] Hueglin C, Gehrig R, Baltensperger U, Gysel M, Monn C, Vonmont H. Chemical characterisation of PM2.5, PM10 and coarse particles at urban, near-city and rural sites in Switzerland. Atmospheric Environment 2005; 39: 637–651.

[5] Lonati G, Giugliano M, Butelli P, Romele L, Tardivo R. Major chemical components of PM2.5 in Milan (Italy). Atmospheric Environment 2005; 39: 1925–1934.

[6] Querol X., Alastuey A., Rodriguez S., Plana F., Ruiz C.R., Cots N. et al PM10 and PM2.5 source apportionment in the Barcelona Metropolitan area, Catalonia, Spain. Atmospheric Environment 2001; 35: 6407–6419.

[7] Pitchford M, Malm W. Development and applications of a standard visual index. Atmospheric Environment 1994; 28(5): 1049–1054.

[8] Malm CW. Introduction to Visibility. Fort Collins, CO: Cooperative Institute for Research in the Atmosphere; 1999.

[9] Bishop I, Rohrmann B. Subjective responses to simulated and real environments: A comparison. Landscape and Urban Planning 2003; 65(4): 261–277.

[10] Bishop ID, Ye WS, Karadaglis C. Experiential approaches to perception response in virtual worlds. Landscape and Urban Planning 2001; 54(1–4): 117–125.

[11] Ode A, Fry G, Tveit MS, Messager P, Miller D. Indicators of perceived naturalness as drivers of landscape preference. Journal of Environmental Management 2009; 90(1): 375–383. 10.1016/j.jenvman.2007.10.013 18280633

[12] Davidson K, Deck L, Houle C. Assessing people opinions on visibility impairment due to air pollution: Summary report Research Triangle Park, NC: U.S. Environmental Protection Agency; 2001.

[13] Schulze WD, Brookshire DS, Walther E, Kelley K. Methods development for environmental control benefits assessment, Vol. X: The benefits of preserving visibility in the national parklands of the Southwest Washington, DC: U.S. Environmental Protection Agency, Office of Research and Development; 1981.

[14] Rahmatian M. Extensions of the disaggregate bid experiment: Variations in framing. Journal of Environmental Management 1986; 22(3): 191–202.

[15] Tolley G, Randall A, Blomquist G, Fabian R, Fishelson G, Frankel A et al Establishing and valuing the effects of improved visibility in Eastern United States Washington, D.C.: U.S. Environmental Protection Agency; 1984.

[16] Rae D. Benefits of improving visual air quality in Cincinnati: Results of a contingent valuation survey Palo Alto, CA: Electric Power Research Institute; 1984.

[17] Rowe RD, d'Arge R, Brookshire D. An Experiment on the economic value of visibility. Journal of Environmental Economics and Management 1980; 7(1): 1–19.

[18] Rowe RD, Chestnut LG. Valuing environmental commodities: Revisited. Land Economics 1983; 59(4): 404–410.

[19] McFaland KK, Malm W, Molenar J. An examination of methodologies and social indicators for assessing the value of visibility In: Chestnut RDRaLG editor. Managing Air Quality and Scenic Resources at National Parks and Wilderness Areas. Boulder, Colorado: Westview Press; 1983.

[20] Council of Europe. Presentation of the European Landscape Convention. Strasbourg: Council of Europe; 2003.

[21] Lothian A. Landscape and the philosophy of aesthetics: Is landscape quality inherent in the landscape or in the eye of the beholder? Landscape and urban planning 1999; 44(4): 177–98.

[22] Meinig DW. The beholding eye: Ten versions of the same scene. Landscape Architecture 1976; 66: 47–54.

[23] Gao J, Chai FH, Wang T, Wang SL, Wang WX. Particle number size distribution and new particle formation: New characteristics during the special pollution control period in Beijing. Journal of Environmental Sciences 2012; 24(1): 13–20. 10.1016/s1001-0742(11)60725-0 22783611

[24] Chai FH, Gao J, Chen ZX, Wang SL, Zhang YC, Zhang JQ et al Spatial and temporal variation of particulate matter and gaseous pollutants in 26 cities in China. Journal of Environmental Sciences 2014; 26: 75–82. 10.1016/s1001-0742(13)60383-6 24649693

[25] Steg L, Ven Den Berg AE, De Groot JIM. Environmental Psychology: An Introduction. West Sussex, UK; Wiley-Blackwell; 2013.

[26] Chestnut LG, Dennis RL. Economic benefits of improvements in visibility: Acid rain provisions of the 1990 clean-air act amendments. Journal of the Air & Waste Management Association 1997; 47(3): 395–402. 29081291 10.1080/10473289.1997.10464437

[27] Hanemann WM. Valuing the environment through contingent valuation. The Journal of Economic Perspectives 1994; 8(4): 19–43.

[28] Carson RT, Hanemann WM. Contingent valuation In: Maler KG, Vincent JR, editors. Handbook of Environmental Economics. North-Holand; 2003.

[29] Sikorski C, Luppa M, Schomerus G, Werner P, König H-H, Riedel-Heller SG. Public Attitudes towards Prevention of Obesity. Plos ONE 2012; 7(6): e39325 10.1371/journal.pone.0039325 22723996 PMC3378564

[30] Wang Y, Zhang Y-S. Air quality assessment by contingent valuation in Ji'nan, China. Journal of Environmental Management 2008; 90(2): 1022–1029. 10.1016/j.jenvman.2008.03.011 18468772

[31] Milfont TL. Psychology of environmental attitudes: A cross-cultural study of their content and structure. Unpulished doctoral dissertation. Auckland, New Zealand: University of Auckland; 2007.

[32] Dunlap RE, Van Liere KD. The “new environmental paradigm”. Journal of Environmental Education 1978; 9: 10–19.

[33] Weigel R, Weigel J. Environmental concern: the development of a measure. Environment and Behavior 1978, 10: 3–15.

[34] Maloney MP, Ward MP. Ecology: let’s hear it from the people. An objective scale for measurement of ecological attitudes and knowledge. American Psychologist 1973; 28: 583–586.

[35] Milfont TL, Duckitt J. The structure of environmental attitudes: A first- and second-order confirmatory factor analysis. Journal of Environmental Psychology 2004; 24: 289–303.

[36] Grob A. A structural model of environmental attitudes and behavior. Journal of Environmental Psychology 1995; 15: 209–220.

[37] Bodur M, Sarigollu E. Environmental sensitivity in a developing country: Consumer classification and implications. Environment and Behavior 2005; 37(4): 487–510.

[38] Shen J, Saijo T. Reexamining the relations between socio-demographic characteristics and individual environmental concern: Evidence from Shanghai data. Journal of Environmental Psychology 2008; 28: 42–50.

[39] Schaffer BS, Riordan CM. A review of cross-cultural methodologies for organizational research: A best- practices approach. Organizational Research Methods 2003, 6: 169–214.

[40] Converse JM, Presser S. Survey Questionnaires: Hand Crafting the Questionnaire. Beverly Hills, CA: Sage; 1986.

[41] Farh J-L, Cannella AA, Lee C. Approaches to scale development in Chinese management research. Management and Organization Review 2006; 2(3): 301–318.

[42] Baron RM, Kenny DA. The moderator-mediator variable distinction in social psychological research: conceptual, strategic, and statistical considerations. Journal of Personality and Social Psychology 1986; 51(6):1173–1182. 3806354 10.1037//0022-3514.51.6.1173

[43] Xinhua. China to suffer more from smog. Chinadaily 2013; 1: 31 Available: http://www.chinadaily.com.cn/china/2013-01/31/content_16188944.html

[44] Hobbs JE. Liability and traceability in agri-food supply chain In: Ondersteijn CJM, Wijnands JHM, Huirne RBM, van Kooten O., editors. Quantifying the agri-food supply chain. Netherlands: Springer; 2006.

[45] Zander KK, Parkes R, Straton A, Garnett ST. Water Ecosystem Services in Northern Australia—How Much Are They Worth and Who Should Pay for Their Provision? PLoS ONE 2013; 8(5): e64411 10.1371/journal.pone.0064411 23717611 PMC3663746

